# Two new European long-legged hoverfly species of the *Eumerusbinominatus* species subgroup (Diptera, Syrphidae)

**DOI:** 10.3897/zookeys.858.34663

**Published:** 2019-07-01

**Authors:** Ana Grković, John Smit, Snežana Radenković, Ante Vujić, Jeroen van Steenis

**Affiliations:** 1 Department of Biology and Ecology, University of Novi Sad, Trg Dositeja Obradovića 2, 21000 Novi Sad, Serbia University of Novi Sad Novi Sad Serbia; 2 European Invertebrate Survey – the Netherlands, PO Box 9517, 2300 RA, Leiden, The Netherlands European Invertebrate Survey – the Netherlands Leiden Netherlands; 3 Research Associate Naturalis Biodiversity Center, Leiden. Hof der Toekomst 48, 3823 HX Amersfoort, The Netherlands Research Associate Naturalis Biodiversity Center Leiden Netherlands

**Keywords:** *Binominatus* subgroup, *
Eumerus
*, hoverflies, identification key, long-legged syrphids, *tricolor* group, Turano-Mediterranean distribution

## Abstract

*Eumerus* Meigen (Diptera, Syrphidae) is one of the most speciose hoverfly genera in Europe, with several species groups recognized within. As part of the *tricolor* group of species, a subgroup of long-legged representatives stands out. We name it *Eumerusbinominatus* subgroup and provide descriptions for two new European species which belong to this subgroup: *E.grallator***sp. nov.** from mainland Spain and *E.tenuitarsis***sp. nov.** from Lesvos and Evros, Greece. A key for all five recognized species of the *binominatus* subgroup is provided.

## Introduction

The genus *Eumerus* Meigen (Merodontini) is one of the most species-rich hoverfly genera in the World. There are over 300 species known (Evenhuis and Pape 2019) and new ones are described on an almost yearly basis. The European fauna is fairly well known, although even here new species are regularly found, especially in the Mediterranean. Since 2010 no less than 17 species have been described from southern Europe and the adjacent Middle East (Ricarte et al. 2012, 2018; Grković et al. 2015, 2017, 2019; Markov et al. 2016; Smit et al. 2017; Steenis et al. 2017; Chroni et al. 2018) and many more await description.

The members of *Eumerus* recorded in Southeast Europe belong to the following groups, identified on the basis of molecular markers in Chroni et al. (2017) as well as on the basis of morphological similarity: *tricolor* (Chroni et al. 2017; Grković et al. 2017), *strigatus* with subgroup *bactrianus* (Speight et al. 2013; Grković et al. 2017, 2019), *clavatus* (Grković et al. 2017), *minotaurus* (Chroni et al. 2018), *barbarus* (van Steenis et al. 2017), *olivaceus*, *ornatus*, *obliquus* (Smit et al. 2017), *basalis* and *pulchellus*. The *Eumerustricolor* species group, defined by Chroni et al. (2017) based on DNA sequencing and described by Grković et al. (2017) displays a wide spectrum of species, but is clearly separated from the other members of the genus by a set of apomorphic characters, including a radially wrinkled basoflagellomere and a fossette clearly expressed, most often with partially to completely red abdominal tergites, but also includes species without red markings. This group makes up about 30% of all *Eumerus* species in the Mediterranean Region.

**Figure 1.**
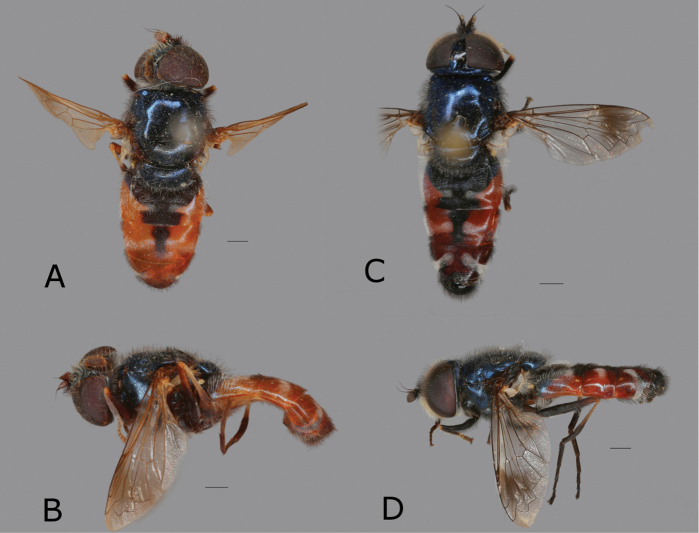
*Eumerusbinominatus*, male holotype of *E.maculipennis***A** dorsal view **B** lateral view. *Eumerustadzhikorum*, male, Kazachstan **C** dorsal view **D** lateral view. Scale bar: 1.0 mm.

In this paper we add yet another two European species to the list of *tricolor* group, one from the western Mediterranean and one from the eastern part of it. Both species belong to a species subgroup not previously recorded from the western Palearctic, characterized by slender elongated legs in the male, which is a unique feature within the family of Syrphidae, a long pilose thorax, pilose eyes and a stout abdomen. We included these species into the *binominatus* subgroup named after the Asian long-legged species *E.binominatus* Hervé-Bazin, 1923 (Fig. 1A, B), first described by Becker as *E.maculipennis*, a name preoccupied by Bezzi (1915) for an African species from the *ornatus* group.

## Material and methods

The characters used in the key, descriptions, and drawings follow the terminology established by Thompson (1999). Terminology referring to male genitalia follows Doczkal (1996) and Hurkmans (1993). Color characters are described from dry-mounted specimens. Male genitalia were stored in microvials containing glycerol after clearing in warm 10% potassium hydroxide (KOH) for a few minutes and neutralising in acetic acid for 5–10 seconds. Label information is given in quotes with the lines separated by a slash ‘/’, additional information is provided in square brackets.

The drawings and part of the figures were created using photographs taken with a Leica DFC 320 (Wetzlar, Germany) camera attached to a Leica MZ16 binocular stereomicroscope and then processed in Adobe Photoshop CS3 v10.0 (Adobe Systems, San Jose, CA, USA). The figures of *Eumerusbinominatus* and *E.tadzhikorum* were created using photographs taken with a Canon EOS D6 equipped with a Canon MP-E 65 macro zoom lens. Several photos for each figure were processed with Zerene Stacker and further edited with the Photoshop program GIMP 2.8.22.

The distribution map was created in Adobe Illustrator CS6 V 16.0.0 software (Adobe Systems, San Jose, CA, USA).

The following acronyms for museums and entomological collections are used in the text:

**AEPC** A. van Eck private collection, Tilburg, The Netherlands

**CEUA** Colección Entomológica de la Universidad de Alicante, CIBIO-Alicante University, Spain

**CSCA** California State Collection of Arthropods, Sacramento, California, USA

**DDPC** D. Doczkal private collection, Malsch, Germany

**FSUNS** University of Novi Sad, Department of Biology and Ecology, Novi Sad, Serbia

**NBC** Natural Biodiversity Center, Leiden, The Netherlands

**SBPC** S. Bot private collection, Haren, The Netherlands

**USNM** United States National Museum of Natural History, Smithsonian Institution, Washington, United States

**ZISP** Zoological Museum, Russian Academy of Sciences, St. Petersburg, Russia

**ZMHU** Museum für Naturkunde der Humboldt Universität zu Berlin, Berlin, Germany

## Results

### Taxonomy

#### Family Syrphidae

##### Subfamily Eristalinae

###### Tribe Merodontini

####### Genus *Eumerus* Meigen, 1822

######## Species group *tricolor*

######### Species subgroup binominatus

Taxon classificationAnimaliaDipteraSyrphidae

########## Diagnosis.

Eyes densely whitish, pilose. Basoflagellomere relatively small, only about twice size of pedicel, oval to squarish in shape, with only a few short radial wrinkles. Male eyes holoptic or narrowly dichoptic. Abdomen short and stout (Fig. 4C). Posterior lobe of surstylus simple, well developed.

########## Remarks.

The *Eumerusbinominatus* subgroup shares all characters of the *tricolor* group (Grković et al. 2017) but can easily be recognized within this group by the extremely long and slender legs in males, especially obvious in the metaleg, where the width of the widest part of metafemur is equal or less than one fifth of the length of the metafemur (Fig. 4D–F). *Eumerusniveitibia* is a Mediterranean species from the *tricolor* group which shares several characters with members of *binominatus* subgroup, e.g. long pilosity on eyes and thorax, similar heart-shaped abdomen with large white pollinose maculae on tergites, but it is clearly differentiated by the long eye-contiguity in male, metafemur clearly thickened and by characteristic snow-white pilosity dorsally on metatibia. Furthermore, the posterior lobe of the surstylus in *E.niveitibia* is much smaller than in *E.binominatus* species subgroup. Females of *E.niveitibia* are similar in appearance with the females of *binominatus* subgroup, but can be differentiated by a slenderer metafemur and characteristic curvature on the metatibia in the *binominatus* subgroup females.

*E.selevini* Stackelberg, 1949 is a middle-Asian species similar to the *binominatus* subgroup, based on the slender metafemur. The head of this species is very similar to that in *E.binominatus* and *E.tadzhikorum* but with smaller, equilateral ocellar triangle, placed medially on vertex, which is in the other two species large, elongated and placed closer to the upper eye margins. It differs by the normal shaped metatarsus, not elongated as in *binominatus* subgroup; elongate abdomen in comparison to the length of head and thorax together and with a characteristic lateral notch in the second metatarsal segment; the pilosity on the thorax is very short in *E.selevini* in contrast to species in the *binominatus* subgroup which makes this species easily recognizable.

The following species belong to the *binominatus* subgroup:

*E.binominatus* Hervé-Bazin, 1923 (Fig. 1A, B)

= *E.maculipennis* Becker, 1921 preocc. Bezzi, 1915

*E.grallator* sp. nov. (Fig. 3A, B)

*E.longitarsis* Peck, 1979

*E.tenuitarsis* sp. nov. (Fig. 3C, D)

*E.tadzhikorum* Stackelberg, 1949 (Fig. 1C, D)

######### Eumerus binominatus

Taxon classificationAnimaliaDipteraSyrphidae

Hervé-Bazin, 1923

Fig. 1A, B

########## Notes.

This species was originally described by Becker (1921) as *E.maculipennis* from Transcaspia (south part of Kazakhstan). Hervé-Bazin (1923) revealed this name as a junior homonym of *Eumerusmaculipennis* Bezzi, 1915 from Nigeria and named Becker’s species *E.binominatus*. The holotype is held in ZMHU and has been examined.

########## Material examined.

Holotype ♂ *Eumerusmaculipennis* Becker, 1921: “Transkaspien / 57442”, “*maculipennis* / Beck / det Becker”, “Holotypus” [red label], “Zool. Mus. / Berlin”, “Holotype ♂ / *Eumerusmaculipennis* / Becker, 1921 / det. J. van Steenis, 2016, (ZMHU).

########## Diagnosis.

**Figure 2.**
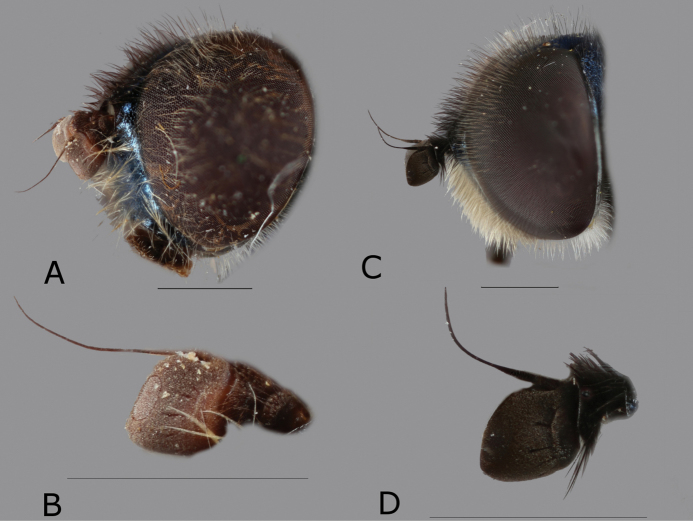
*Eumerusbinominatus*, Male holotype of *E.maculipennis***A** head, fronto-lateral view **B** antenna, lateral view. *Eumerustadzhikorum*, male, Kazachstan **C** head lateral view **D** antenna lateral view. Scale bar: 1.0 mm.

Male eyes separated by width of ocellus. Face black, covered in whitish pilosity with few black pilosities above antennae (Fig. 2A). Antenna brown-red and slightly higher than long (Fig. 2B). Wing with a dark spot (Fig. 1B). Metafemur with row of about 7 rather long black setae, which are about 1/2 as long as width of metafemur. Abdomen partly red (Fig. 1A).

This species is similar to *E.tadzhikorum* but differentiated by the shape and color of the basoflagellomere.

######### Eumerus grallator

Taxon classificationAnimaliaDipteraSyrphidae

Smit sp. nov.

http://zoobank.org/F77091EF-7681-4560-8370-447DDB792687

**Figure 3.**
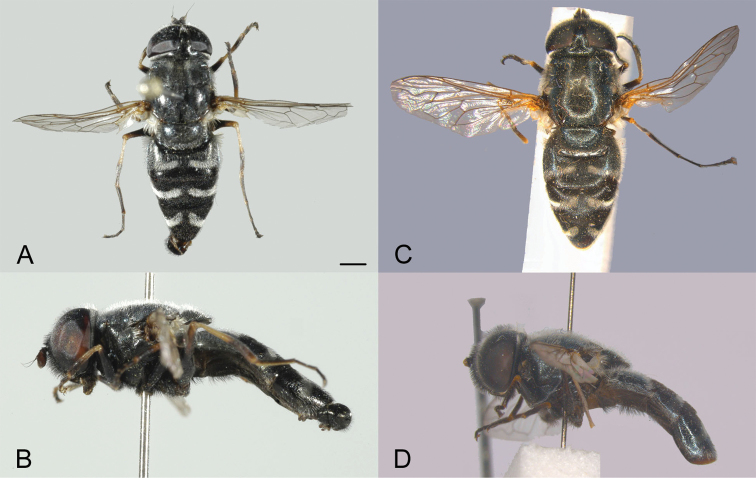
*Eumerusgrallator* sp. nov., male holotype **A** dorsal view **B** lateral view. *Eumerustenuitarsis* sp. nov. male holotype **C** dorsal view **D** lateral view. Scale bar: 1 mm.

**Figure 4.**
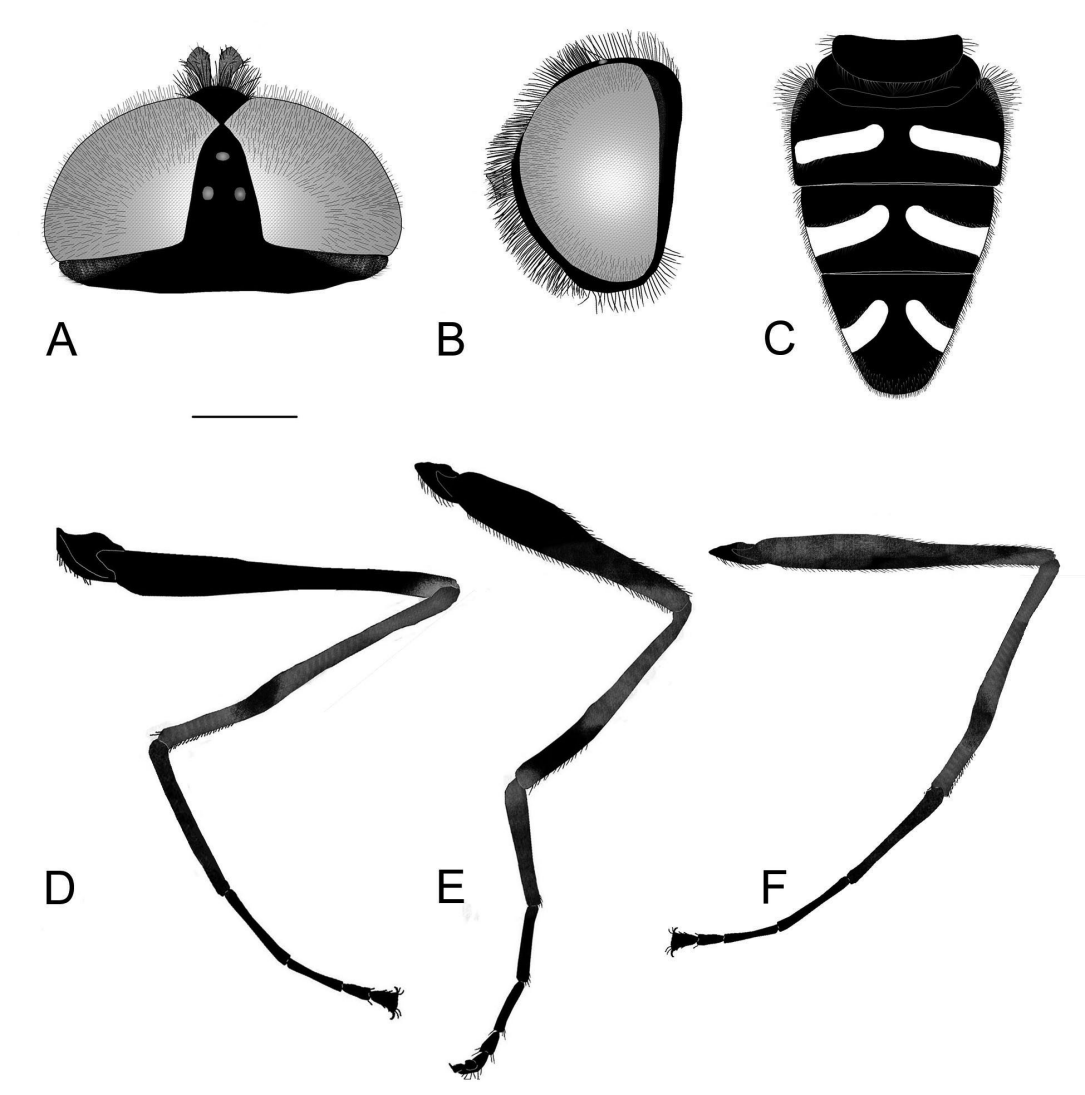
*Eumerustenuitarsis* sp. nov., male **A** head, dorsal view **B** head, lateral view **C** abdomen **D** metaleg, lateral view. *Eumerusgrallator* sp. nov., male **E** metaleg, lateral view. *Eumeruslongitarsis*, male **F** metaleg, lateral view. Scale bar: 1 mm.

**Figure 5.**
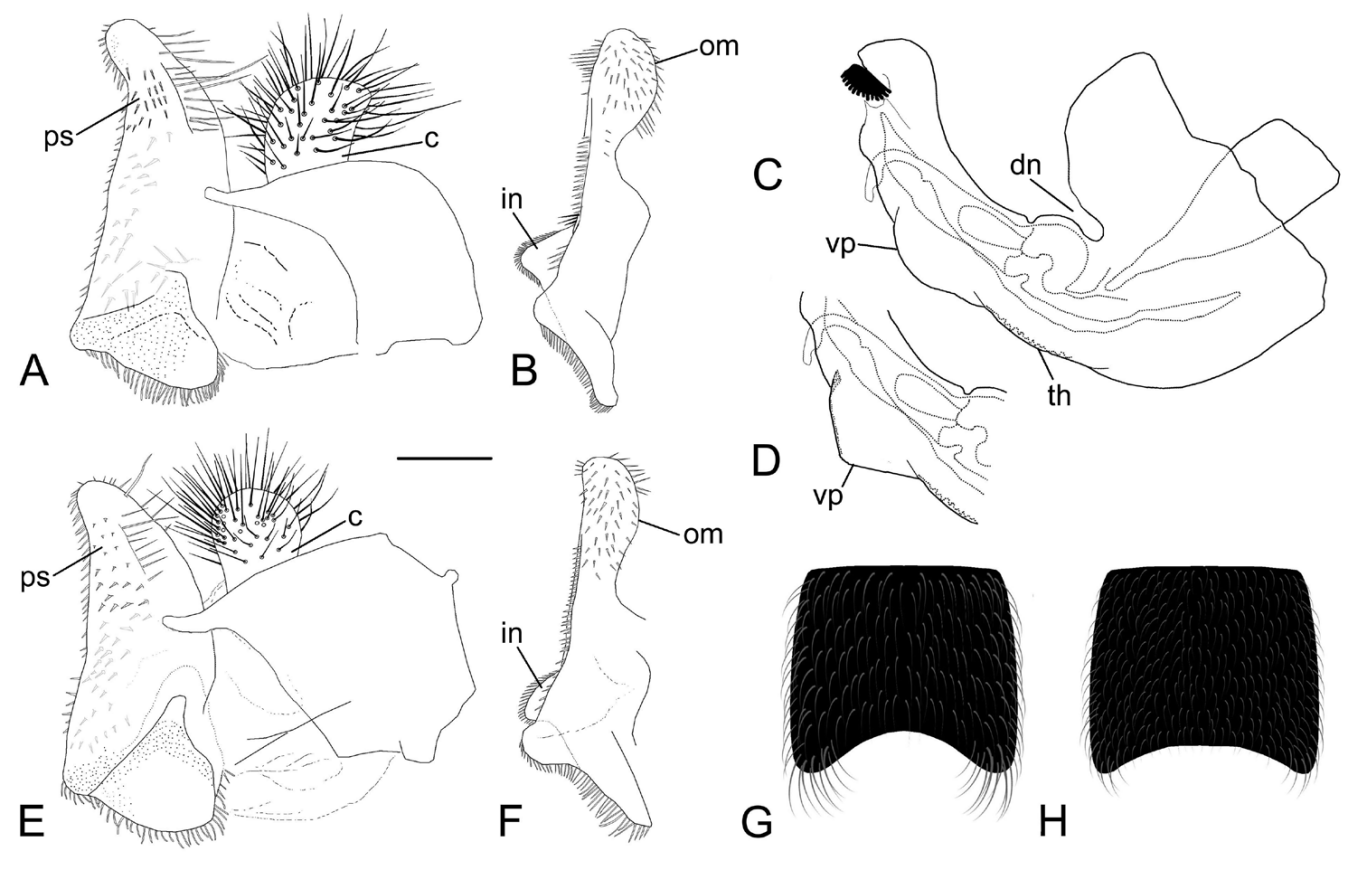
*Eumerusgrallator* sp. nov., male **A** epandrium, lateral view **B** surstyle lobe, ventral view **D** medial part of hypandrium, lateral view **G** fourth sternum. *Eumerustenuitarsis* sp. nov., male **C** hypandrium, lateral view **E** surstyle lobe, lateral view **F** epandrium, ventral view **H** fourth sternum. **Abbreviations**: **c** – cercus, **dn** – dorsal notch of hypandrium, **in** – interior accessory lobe of surstyle lobe, **om** – outer margin of posterior surstyle lobe in ventral view, **ps** – posterior lobe of surstylus, **th** – thecal ridge of hypandrium, **vp** – ventral protuberance of hypandrium. Scale bars: 0.2 mm (**A–F**); 0.5 mm (**G, H**).

Figs 3A, B ; 4E ; 5A, B, D, G

########## Type material.

**Holotype.** SPAIN ♂, Castilla la Mancha, Villahermosa. Original label: “España, Castilla / la Mancha, Villahermosa / [UTM] 30S WH19329-88405 / 23.vi.2003. 980 m / leg. J.T. Smit”. The holotype is in good condition with no apparent signs of wear, except for wingtips, which are both damaged. The holotype is deposited in the NBC. **Paratypes**. SPAIN • 1 ♂ same data as for holotype (NBC); 1♀, Andalusia, Los Marines, 600 m, 37°17'05"N 06°22'08"W, 10.vi.2015, leg. J. and I. Smit (NBC); 1♀, Foia Ampla, 1060 m, Agres, Alicante, 3–17.vii.2001, leg. Pérez-Bañon, Marcos-García y Rojo (FSUNS); 1♀, Foia Ampla, 1060 m, Agres, Alicante, 2–16.vii.2002, leg. Pérez-Bañon, Marcos-García y Rojo (CEUA); 1♂, Mas del Parral, 900 m, Bocairent, Valencia, 5–19.vi.2001, leg. Pérez-Bañon, Marcos-García y Rojo (FSUNS); 1♂, “FO: 5335 Spanien / 36°58'29"N, 04°00'59"W / Bosque del Puerto Navazo, Alhama de / 1180 m NN / A; *Thapsiavillosa* / leg. A. Ssymank, 12.06.2003” (DDPC); 1♂, “España, Madrid, Aranjuez / UTM 30T 4484430, 545 m a.s.l. / 17.vi.2015, leg. P.A. Fidalgo” (AEPC); 1♂, “España, Burgos, Peñahorada / UTM 30T 4474705, 910 m a.sl. / 13.vii.2016, leg. P.A. Fidalgo” (AEPC); 2♂ “España, Soria, Herrera de Soria / UTM 30T 4984624 1095 m a.s.l. / 14.vii.2016, leg. P.A. Fidalgo” (AEPC).

########## Diagnosis.

Male. Ocellar triangle isosceles. Basoflagellomere blackish, small, rounded, with one or two short radial wrinkles. Constriction of elongated metafemur located in posterior half (Fig. 4E). Greatest width of metafemur is approximately equal to one fifth of length of metafemur. Metatibia noticeably shorter than metafemur. Abdomen black, without red markings. Ventral margin of hypandrium with medial triangular protuberance (Fig. 5D: vp). Anterior lobe of surstylus with a single pilose row (Fig. 5A).

########## Description.

**Male**. Body length (excluding antenna): 11.5 mm; wing length: 7 mm. *Head*. Eyes separated by the width of an ocellus and covered in dense white pilosity. Eye margins in anterior view almost parallel, slightly broadening ventrally. Face completely black pilose, covered in silver pollinosity, most expressed in middle. Frons, vertical triangle and occiput black; silver pollinosity well expressed along eye margin on frons, on vertex anteriorly and dorsally on occiput behind eye margin, but most distinctive laterally. Ocellar triangle isosceles and predominantly black pilose, becoming intermixed with white pile in front of ocellar triangle and turning predominantly white behind it. Distance from anterior to posterior ocellus same as distance from latter one to upper eye corner. Lower facial margin in lateral view not protruding. Scape and pedicel brown to black. Basoflagellomere dark brown, rounded and slightly longer than broad with one or two short radial wrinkles. Ventral pile of pedicel black, not longer than its depth. *Thorax*. Scutum and scutellum densely punctate, shiny, with a bluish tinge; covered in long dense white pilosity. Two vittae of white pollinosity on scutum faint and thin, hardly reaching base of wings. Pleurae black. Anepisternum entirely white pilose, except for some black pile just behind the anterior spiracle. Anepimeron white pilose with some black pile posteriorly. Katepisternum and katepimeron black pilose. *Wing*. Hyaline with pterostigma about same color as the wing. Vein R_4+5_ slightly curved. Wing covered in microtrichia except for basal cells mostly bare. Costal setae black. Halter blackish. *Legs*. Metaleg slender with all segments very elongated (Fig. 4E). Femora black, yellowish posteriorly, covered in black pilosity. Pro- and mesofemur black with yellowish tips; metafemur black, turning lighter in apical third, becoming orange at apex; slightly thickened in basal half; with a few scattered black setae in apical half. Tibiae white pilose. Pro- and mesotibia mostly black, yellowish in basal third and with yellowish apices; metatibia in basal half yellowish, apical half black turning lighter towards apex. Metatibia slightly thickened apically and slightly curved in apical half (curvature being species-specific in all three species with black abdomen). Tarsi brown to black; basitarsus of metaleg lighter ventrally; metatarsus longer than tibia (Fig. 4E). *Abdomen*. Black, punctate, pilose, tapered (Fig. 3A). Terga 2–3 with pairs of wide white pollinose maculae, slightly obscured towards medial part of terga; tergum 4 with pair of white pollinose maculae with apices upwards. Tergum 2 with long white pile laterally; pilosity adpressed, in area of pollinose maculae white, black on rest of terga. Punctation is visible through pollinose maculae. Genital capsule covered in erect black pilosity. Sterna entirely black pilose; sternum 4 flat with longer pilosity apico-laterally (Fig. 5G). *Terminalia*. (Fig. 5A, B, D). Posterior surstyle lobe simple, beak-like in lateral view, with long strong setae laterally on outer surface (Fig. 5A: ps); in ventral view, outer margin convex, pilose (Fig. 5B: om). Cerci oval, slightly pointed apico-dorsal, uniformly pilose (Fig. 5A: c). Interior accessory lobe of surstyle lobe densely pilose (5B: in). Hypandrium curved, broad with folded thecal ridge near base; medially with triangular protuberance on ventral margin (Fig. 5D: vp), and wide notch dorsally near base. **Female.** Body length (excluding antennae): 11.5 mm; wing length: 7 mm. Similar to male except normal sexual dimorphism and for following differences: *Head*. Entirely white pilose. Basoflagellomere oval, with three to four radial wrinkles. Width of frons in narrower part is less than one fourth of width of head in anterior view. *Thorax*. White pollinose vittae obscured. Pleurae white pilose. Segments of metaleg only slightly elongated. *Abdomen*. Tergum 4 with longer white pile posteriorly. All sterna white pilose.

########## Etymology.

The specific epithet is the Latin word *grallator* meaning “one who walks on stilts”, which refers to the very slender and elongated legs of this species. It should be treated as a noun in apposition.

########## Distribution.

Spain.

########## Remarks.

The male holotype and one male paratype specimens were swept from a stand of some large yellow Apiaceae along a road, in an open park-like landscape of an oak dehesa. Accompanying hoverfly species were *Eristalinustaeniops* (Wiedemann, 1818), *Eristalisarbustorum* (Linnaeus, 1758), *Eumerusbarbarus* (Coquebert, 1804), *E.nudus* Loew, 1848, *Spilomyiadigitata* (Rondani, 1865) and *Xanthogrammamarginale* (Loew, 1854).

######### Eumerus longitarsis

Taxon classificationAnimaliaDipteraSyrphidae

Peck, 1979

Fig. 4F

########## Notes.

This species was described from Tajikistan and is known from Asia Minor and south-central Asia. This species is likely to consist of a complex of closely related species in this region (Doczkal pers. comm.).

########## Material examined.

Holotype ♂ *Eumeruslongitarsis* Peck, 1979: “Tajikistan, Hissar mountains / Takob ravine / Tian Shan h = 1700 m / leg. 23.vii.1976”, “399”, “Holotypus ♂ / *Eumerus* / *longitarsis* Peck” (ZISP).

Additional material: “[Russia] So.[uthern] Primor’e [Primorsky Krai] / Kamenushka / A.Shatalkin [leg.]”, 1♂ (USNM).

########## Diagnosis.

Male. Ocellar triangle equilateral. Face with black pile. Constriction of the elongated metafemur is located in posterior half. Metatarsus remarkably longer than metatibia (Fig. 2F). Anterior lobe of surstylus with multiple rows of long pilosity (see fig. 6 in Peck 1979). Abdomen black, without red markings.

######### Eumerus tadzhikorum

Taxon classificationAnimaliaDipteraSyrphidae

Stackelberg, 1949

**Figure 6.**
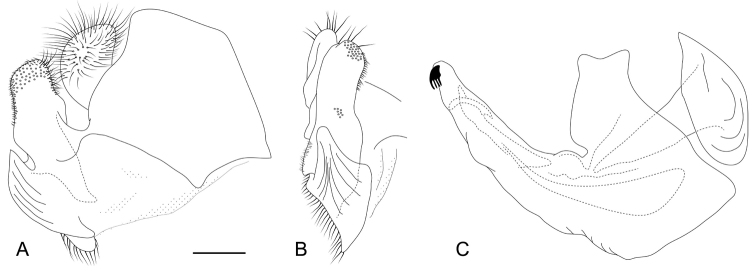
*Eumerustadzhikorum*, male terminalia **A** epandrium, lateral view **B** surstyle lobe, ventral view **C** hypandrium, lateral view. Scale bar: 0.2 mm.

Figs 1C, D ; 2C, D ; 6

########## Notes.

Described from Tajikistan and known from southern Kazakhstan, Kyrgyz, Tajikistan, Turkmenistan and Uzbekistan (Peck 1988). The holotype is held in ZISP and has been examined by the last author. Additional material of *E.tadzhikorum* identified by Stackelberg was studied too.

########## Material examined.

Holotype ♂ *Eumerustadzhikorum* Stackelberg, 1949: “16.VI.[19]44”, “*Eumerus* typ. ’46 / *tadzhikorum* sp. nov. / Stackelberg det.”, ”Holotypus ’49 / *Eumerus* / *tadzhikorum* Stack.” [red label, partly handwritten], ”Lectotypus *Eumerus* / *tadzhikorum* Stack / design. V. Richter” [red label, partly handwritten], (ZISP).

Additional material. Kazakhstan: “KZ Oblast Almaty / Tamgaly 886 m / lat 43.802 lng 75.534 / 8 V 2015 leg. S. Bot”, 1♂ (SBPC); “KAZAKHSTAN 29.V.2001 / SE Chilik 700m / 43°40'N 78°29'E / leg. M. Hauser”, 1♂ (CSCA). Armenia: “Мегри на р. Аракс / Армения / В. Рихтер” with added handwritten “2.5 km B. m. g. / стаиции / 7.V.974”, 1♂ (ZISP).

########## Diagnosis.

Male. Eyes clearly separated by width of basoflagellomere. Wing with a dark spot (Fig. 1C, D). Antenna black and as high as long (Fig. 2D). Metafemur with row of about 7 rather long black setae, which are about 1/3 as long as width of metafemur. Abdomen partly red (Fig. 1C, D). Posterior surstyle lobe anteriorly with fan-like protruding structure, separated by deep incision.

########## Remarks.

This species is extremely similar to *E.binominatus* and it is possibly a subjective junior synonym of this species. The length of the setae on the apico-ventral side of the metafemur seems to vary in number and length. Due to lack of material no conclusion will be drawn here.

######### Eumerus tenuitarsis

Taxon classificationAnimaliaDipteraSyrphidae

Grković & Vujić sp. nov.

http://zoobank.org/4c2cf1d2-c5f5-4e5e-9c2e-562329fdf1f5

**Figure 7.**
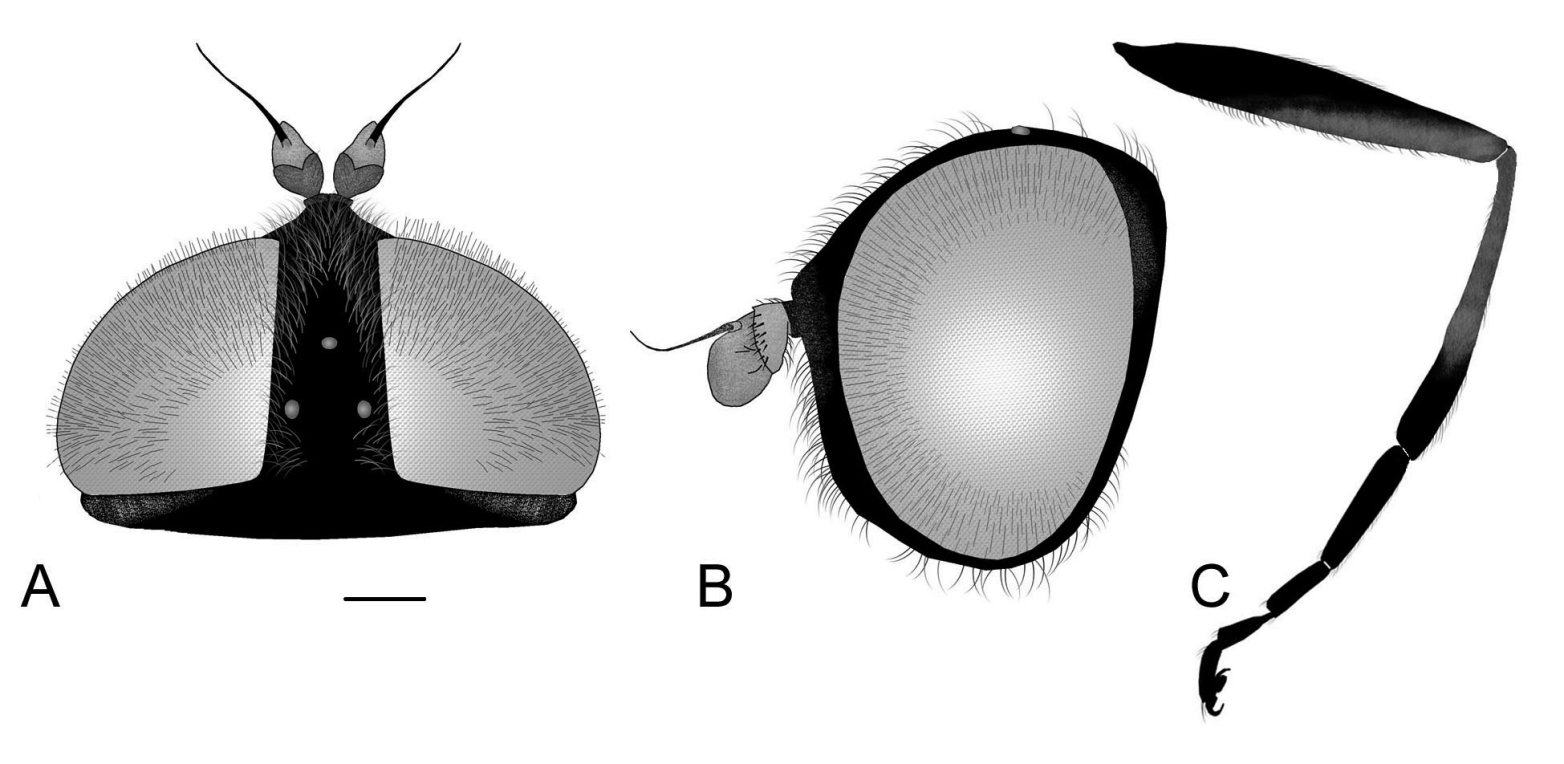
*Eumerustenuitarsis* sp. nov., female **A** head, dorsal view **B** head, lateral view **C** metaleg, lateral view. Scale bar: 0.5 mm.

Figs 3C, D ; 4A–D ; 5C, E, F, H ; 7

########## Type material.

**Holotype**. GREECE • ♂, Lesvos, Agiassos. Original label: “Agiassos, 601 m / Lesbos, Greece / 39°4'16"N / 26°22'23"E / 23.vi.2003 / leg. M. Hull”. **Paratype**. GREECE • 1♀, Evros, Dadia, 26–28.vii.2013, 40,9943N 26.0933E leg. M. Kourtidou (FSUNS).

########## Diagnosis.

Male. Ocellar triangle isosceles (Fig. 4A). Abdomen black, without any red markings (Fig. 3C). Constriction of elongated metafemur is located in anterior half (Fig. 4D). Metatibia with characteristic curvature in posterior half, also well noticeable in female. Ventral margin of hypandrium with oval medial protuberance (Fig. 5C: vp). Anterior lobe of surstylus with a single row of pilosity (Fig. 5E).

########## Description.

**Male**. Body length (excluding antennae): 12 mm; wing length: 8 mm. *Head*. Eyes slightly dichoptic, separated by width of two ommatidia (Fig. 4A); covered in long dense white pilosity. Eye margins in anterior view almost parallel, slightly broadening ventrally. Face completely black pilose, gently pollinose, with distinctive thin line of silvery-white pollinosity in middle, slightly narrower in upper part. Frons, vertical triangle and occiput black; silvery-white pollinosity well expressed along eye margin on frons, on vertex anteriorly and dorsally on occiput behind eye margin, on the posterior margin as a patch, but most distinctive laterally. Ocellar triangle isosceles and predominantly black pilose, becoming intermixed with white pile in front of the ocellar triangle and turning predominantly white behind it. Vertical triangle and occiput with metallic blue reflection. Distance from anterior to posterior ocellus same as distance from latter one to upper eye corner. Lower facial margin in lateral view not protruding (Fig. 4B). In lateral view, white pilosity on eyes make contrast to black long pile on face, reaching one third to half of their length. Scape and pedicel dark colored, almost black. Basoflagellomere lacking in the holotype. Ventral pile of pedicel black, longer than its depth. *Thorax*. Scutum and scutellum densely punctate, covered in long dense white pilosity. Scutum with pair of white vittae of pollinosity, along almost two thirds of scutum length. Scutellum and lateral area of scutum with metallic blue tinge. Pleurae black. Anepisternum predominantly covered in long white pilosity, except behind anterior spiracle with patch of black pilosity. Katepisternum, anepimeron and katepimeron black pilose. *Wing*. Hyaline with pterostigma the same color as wing. Vein R_4+5_ slightly curved. Wing covered in microtrichia except for basal cells mostly bare. Costal setae black. Halter blackish. *Legs*. Metaleg slender with all segments very elongated (Fig. 4D). Femora black with yellowish tips covered in black pilosity; metafemur very narrow in apical half with only few scattered inconspicuous setae. Tibiae white pilose. Pro- and mesotibia mostly black, yellowish in basal third and with yellowish apices; metatibia in the basal half yellowish, the apical half black turning lighter towards apex. Metatibia with characteristic curvature in posterior half (Fig. 4D). Tarsi brown to black; metatarsus longer than tibia. *Abdomen*. Black, punctate, pilose, tapered (Figure 3C; 4C). Terga 2–3 with pairs of wide white pollinose maculae, slightly obscured towards medial part of terga; tergum 4 with pair of white pollinose maculae with apices upwards. Tergum 2 with long white pile laterally; pilosity adpressed, in area of pollinose maculae white, black on rest of terga. Punctures are visible through pollinose maculae. Sterna entirely black pilose; sternum 4 flat with uniformly long pilosity (Fig. 5H). *Terminalia*. (Fig. 5C, E, F). Posterior surstyle lobe simple, beak-like in lateral view, with long strong setae laterally on outer surface (Fig. 5E: ps); in ventral view, outer margin slightly convex, pilose (Fig. 5F: om). Cerci oval (Fig. 5E: c), uniformly pilose. Interior accessory lobe of surstyle lobe densely pilose (Fig. 5F: in). Hypandrium curved, broad with folded thecal ridge near base (Fig. 5C: th); medially with oval protuberance on ventral margin (Fig. 5C: vp) and wide notch dorsally near base (Fig. 5C: dn). **Female**. Body length (excluding antennae): 11 mm; wing length: 7 mm. Similar to male except normal sexual dimorphism and for following characteristics: *Head*. White pilose except on ocellar triangle, with black pilosity. Basoflagellomere oval, dark, reddish anteriorly, with three radial wrinkles (Fig. 7B). Width of the frons in narrower part is narrower than one fourth of width of head in anterior view. (Fig. 7A). *Thorax*. Bluish sheen not noticeable. White pollinose vittae present along almost entire length of scutum. Pleurae white pilose. Segments of metaleg only slightly elongated. Metatibia with characteristic curvature (Fig. 7C). *Abdomen*. Tergum 4 with longer white pile posteriorly. Sterna black pilose except sternum 4 which is covered in white pile.

########## Etymology.

The species name is derived from the Latin words “tenui” and “tarsus” and refers to the extremely long and slender tarsi, especially obvious in the male metalegs.

########## Distribution.

Only known from the holotype and female paratype taken on Lesvos and Evros (Greece) respectively.

**Figure 8.**
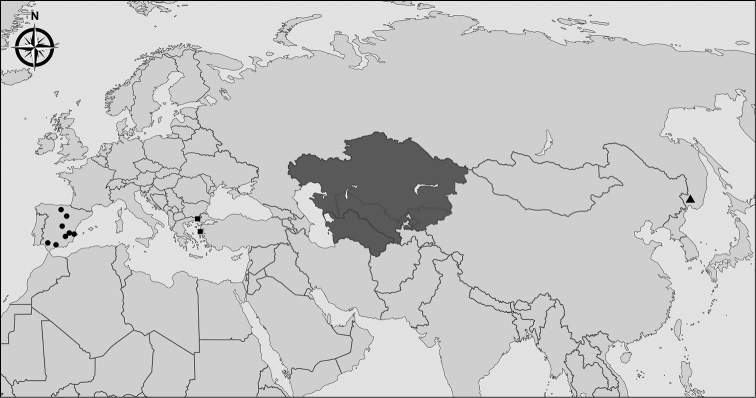
Current known distribution of the species of the *Eumerusbinominatus* subgroup. Black dots stand for *Eumerusgrallator* sp. nov., black triangle for *Eumeruslongitarsis* Peck, 1979 Far East record only, black squares for *E.tenuitarsis* sp. nov. and grey area inferred distribution of Middle Asian species of the *Eumerusbinominatus* subgroup, including *E.binominatus*, *E.longitarsis* and *E.tadzhikorum*.

######## Identification key for the males of the *Eumerusbinominatus* subgroup

Females of the subgroup are very difficult to distinguish morphologically. We have species with red abdomen from middle Asia which can be *E.binominatus* or *E.tadzhikorum* and black species which are in Spain *E.grallator*, in Greece *E.tenuitarsis* and in Middle Asia and Asia minor *E.longitarsis*. The distribution of *E.tenuitarsis* and *E.longitarsis* is not likely to be overlapping in Asia Minor and confusion between the female specimens of these species is not an issue.

**Table:** 

1	Basoflagellomere with more or less marked radially arranged wrinkles and clearly limited fossette. Katepisternum with medial pilosity connecting dorsal and ventral patch of pilosity. Anterior surstyle lobe of epandrium poorly developed	***tricolor* group, 2**
–	Basoflagellomere not wrinkled radially with indistinctly expressed fossette. Katepisternum medially bare, with separated dorsal and ventral patch of pilosity. Anterior surstyle lobe of epandrium well developed	**other *Eumerus* groups (not treated here)**
2	Segments of metaleg extremely elongated, especially metatarsus as long as or longer than metafemur (Fig. 4D–F)	***binominatus* subgroup, 3**
–	Segments of metaleg not extremely elongated, metatarsus about 2/3–3/4 of length of metafemur	**other *Eumerus* species from *tricolor* group (not treated here)**
3	Terga with red markings laterally (Fig. 1). Eyes separated for a large distance. Face white pilose. Wing with a dark spot	**4**
–	Terga completely black (Fig. 3). Eyes touching in one point or very slightly separated by distance of few ommatidia. Face black pilose. Wing without a dark spot	**5**
4	Basoflagellomere brown-red and slightly higher than long (Fig. 2B)	***E.binominatus* Hervé-Bazin**
–	Basoflagellomere black, as high as long (Fig. 2D)	***E.tadzhikorum* Stackelberg**
5	Greatest width of metafemur is approximately equal to one fifth of length of metafemur (Fig. 4E, F). Metatibia noticeably shorter than metafemur	**6**
–	Greatest width of metafemur is approximately equal to one eighth of length of metafemur (Fig. 4D). Metatibia about same length or very slightly shorter than metafemur. Metatibia with characteristic curvature	***E.tenuitarsis* sp. nov.**
6	Metatarsus noticeably longer than metatibia and metafemur (Fig. 4F)	***E.longitarsis* Peck**
–	Metatarsus approximately the equally long as metatibia and metafemur (Fig. 4E)	***E.grallator* sp. nov.**

## Discussion

The *Eumerusbinominatus* subgroup is a group of long-legged species sharing all diagnostic characters with *E.tricolor* group (Chroni et al. 2017; Grković et al. 2017). The main characteristic for the species subgroup is the extremely thin and elongated legs which is an uncommon character for the family as a whole. The herein described species belonging to the *binominatus* subgroup share some characters with the two most similar species from the *tricolor* group – *Eumerusniveitibia* Becker, 1921 and *E.azabense* Ricarte & Marcos-García, 2018. Those characters are shape of abdomen and wide pollinose maculae on the terga, type of pilosity, shape of male epandrium and the presence of a folded thecal ridge on the hypandrium (Fig. 3C).

The remarkable long legs, especially conspicuous in males, could be behaviourally evolved or be part of a form of mimicry (Zimmer et al. 2003). In several hoverfly genera like *Platycheirus* and *Eumerus* (Dziock 2002; Reemer et al. 2009) the males use their legs in signalling for female attraction. In other genera the legs are used as part of the mimicry, like in *Spilomyia* which wave their prolegs in front of their head imitating the long antennae of Hymenoptera (van Steenis 2000; Penny et al. 2014) or in *Sphegina* which have their long metalegs hanging down in flight recalling sphecid wasps (Hippa et al. 2015). The remarkable long legs in the *binominatus* subgroup can have either of these functions and behavioural studies should clarify this in the future.

As shown here, the Turano-Mediterranean region represents a diversity center for the *binominatus* subgroup. The species of the *binominatus* subgroup are, however, not entirely restricted to this region given that we have one specimen of *E.longitaris* from the Russian Far East. This disjunct Turano-Mediterranean distribution is already recorded in several insect orders, in Acari and also in vipers (Ribera and Blasco-Zumeta 1998; Sanmartín 2003; Vujić et al. 2011; Ferchaud et al. 2012; García-Vázquez et al. 2016). It has also been discussed in the genus *Eumerus* within the *bactrianus* subgroup whose species exhibiting disjunct distribution between western and eastern Mediterranean regions extending to the Asiatic steppes to the east (Grković et al. 2019). Some authors discussed whether the Turano-Mediterranean distribution pattern in birds and beetles originate from dispersal or vicariant events (Voelker 1999; Sanmartín 2003). In the genus *Eumerus* it is unclear if the appearance of the geographic barrier occurred after the origin of the *binominatus* subgroup and thus would classify as vicariant event. The last ice age can be such an event separating the western and eastern Mediterranean and causing the speciation process forming *E.grallator* sp. nov. and *E.tenuitarsis* sp. nov. Further research including additional sampling in the Turanian area and DNA analysis could clarify this issue.

## Supplementary Material

XML Treatment for binominatus

XML Treatment for Eumerus binominatus

XML Treatment for Eumerus grallator

XML Treatment for Eumerus longitarsis

XML Treatment for Eumerus tadzhikorum

XML Treatment for Eumerus tenuitarsis
